# Targeted exome sequencing for mitochondrial disorders reveals high genetic heterogeneity

**DOI:** 10.1186/1471-2350-14-118

**Published:** 2013-11-11

**Authors:** Jeana T DaRe, Valeria Vasta, John Penn, Nguyen-Thao B Tran, Si Houn Hahn

**Affiliations:** 1 Transgenomic, Inc, 5 Science Park, New Haven, CT 06511, USA; 2 Seattle Children’s Hospital Research Institute, 1900 9th Ave, Seattle, WA 98101, USA; 3 Department of Pediatrics, Division of Genetic Medicine, University of Washington, School of Medicine/Seattle Children’s Hospital, 4800 Sand Point Way, Seattle, WA 98105, USA

**Keywords:** Mitochondrial disorder, Respiratory chain complexes, Electron transport chains, Next-generation sequencing, Targeted exome

## Abstract

**Background:**

Mitochondrial disorders are difficult to diagnose due to extreme genetic and phenotypic heterogeneities.

**Methods:**

We explored the utility of targeted next-generation sequencing for the diagnosis of mitochondrial disorders in 148 patients submitted for clinical testing. A panel of 447 nuclear genes encoding mitochondrial respiratory chain complexes, and other genes inducing secondary mitochondrial dysfunction or that cause diseases which mimic mitochondrial disorders were tested.

**Results:**

We identified variants considered to be possibly disease-causing based on family segregation data and/or variants already known to cause disease in twelve genes in thirteen patients. Rare or novel variants of unknown significance were identified in 45 additional genes for various metabolic, genetic or neurogenetic disorders.

**Conclusions:**

Primary mitochondrial defects were confirmed only in four patients indicating that majority of patients with suspected mitochondrial disorders are presumably not the result of direct impairment of energy production. Our results support that clinical and routine laboratory ascertainment for mitochondrial disorders are challenging due to significant overlapping non-specific clinical symptoms and lack of specific biomarkers. While next-generation sequencing shows promise for diagnosing suspected mitochondrial disorders, the challenges remain as the underlying genetic heterogeneity may be greater than suspected and it is further confounded by the similarity of symptoms with other conditions as we report here.

## Background

Patients with mitochondrial diseases present with widely variable phenotypes that can affect any organ, at any age. This clinical variability and a lack of reliable diagnostic tests can make diagnosing these disorders very challenging. Currently, the diagnosis relies upon the enzymatic analysis of respiratory chain complexes (RCC) in muscle biopsy tissues [1,2]. Unfortunately, clinical laboratories have considerable differences in their RCC assay protocols and methods of interpretation, leading to inaccurate diagnoses that can affect the quality of patient care [3-5].

Recently, targeted next-generation sequencing (NGS) has been utilized to diagnose patients with several types of disorders [6-11]. For mitochondrial disease, NGS can be used not only to examine genes that have been classically considered to cause disease, such as RCC subunits and assembly factors, but also to analyze genes that are suspected to cause mitochondrial dysfunction, or genes causing conditions that mimic mitochondrial disease [12]. Over 200 nuclear genes have been identified that cause mitochondrial disease, and the list continues to grow as over 1500 genes have been identified controlling mitochondrial structure and function [13,14]. We recently explored a panel of ~900 known and candidate disease genes for 26 patients with known or suspected mitochondrial disorders [15]. This study was successful in demonstrating both the analytical sensitivity and the clinical utility of NGS by diagnosing several of the patients included. Here, we utilize a smaller gene panel (447 genes) which includes 364 known genes causing mitochondrial disease or similar disease, and 83 candidate genes expected to be involved in critical mitochondrial functions, such as RCC subunits and tRNA synthetases. This gene panel was offered to physicians as a commercially available clinical diagnostic assay through Transgenomic, Inc. The samples were processed, analyzed, and interpreted at a CLIA-approved laboratory at Seattle Children’s Research Institute, and the final report was provided through Transgenomic, Inc. Here, we present data from the first 148 patients.

## Methods

### Patients

148 patients were referred by experts in mitochondrial medicine, and all patients had diagnosed or suspected mitochondrial disorders according to the physicians. Limited clinical information was provided for the patients with the test requisition, and the clinical presentations that were provided varied widely. The most common clinical findings included developmental delay and hypotonia, and other common findings are listed (Table 1). Not enough clinical and laboratory information were provided to independently determine if patients fulfilled Modified Walker Criteria for diagnosis of mitochondrial disorders [16], however about 36% of patients did have abnormal RCC enzymes activity and/or abnormal muscle pathology (Table 1). Some patients had undergone previous genetic testing, including mitochondrial DNA (mtDNA) sequencing and single nuclear gene testing. In the few cases where results were provided for these tests, they were either considered negative or contained variants of unknown significance. The majority of patients were under the age of 18 years (83%) and the age range was <1 year to 68 years. Parental samples were also submitted following the release of the proband’s report as recommended to further elucidate the significance of variants found. Written Informed consent was obtained from all patients, or their parents on the behalf of the patients under 18 years of age, when testing were ordered by the patient’s physician. The Institutional Review Board at Seattle Children’s Hospital approved the study (#14631).

**Table 1:** Clinical and laboratory characteristics of patients

**Clinical phenotype**	**Percentage (%)**
Developmental delay	53
Abnormal RCC enzymes activity	21
Abnormal muscle biopsy	15
Hypotonia	30
Seizures	28
Gastrointestinal dysmotility	26
Fatigue/exercise intolerance	17
Neuropathy	8
Ataxia	7
Dystonia	4

### DNA capture and sequencing

A DNA library was prepared for each sample using an Illumina Genome DNA Sample preparation kit (Illumina, San Diego, CA). The exons of the genes of interest (Additional file 1: Table S1) were captured by in-solution hybridization using custom made probes (SureSelect, Agilent, Santa Clara, CA).

The gene panel run from June 2011 to August 2011 consisted of 418 genes, and then was expanded to 447 genes in September 2011. These genes include all of the nuclear-encoded structural components of the mitochondrial respiratory chain complexes (n = 89), as well as mitochondrial respiratory chain complexes assembly factors (n = 29), mitochondrial carriers (n = 17), genes for mitochondrial DNA synthesis, transcription, translation, mitochondrial biogenesis and dynamic (n = 71), genes for mitochondrial enzymes (n = 114), and other genes that may affect mitochondrial function secondarily or that cause similar clinical phenotypes (n = 127) (Additional file 1: Table S1). Some genes are overlapping in the categories. mtDNA sequencing was not included in this test as it is available as a separate and less costly test. Sequencing was performed by a Genome Analyzer IIx instrument (Illumina, San Diego, CA) using single-end reads and one sample per lane on the 8-lanes flow-cell.

### Data analysis

Reads were aligned using Burrows-Wheeler Aligner (BWA) [17]. Data were analyzed with the Genome Analysis Toolkit (GATK) (Broad Institute, Cambridge, MA) Unified Genotyper (version 1.0.4013) and Variant Filtration Walker to filter the variants that meet quality control requirements. Insertion and deletions were analyzed with Dindel (GATK version 1.0.5336) [18]. Variants found within the targeted regions (exons +/− 20 bp on either side of the exon) were further evaluated for their possible clinical significance by cross-referencing to dbSNP and the 1000 genomes browser. An internal database of polymorphisms was also used during this analysis. For variants in genes with autosomal dominant (AD) and X-linked (XL) disease inheritance, we used the minor allele frequency (MAF) cut-off of 0.2%, and for variants in genes with autosomal recessive disease (AR) inheritance, we used the MAF cut-off of 0.5%. Variants that exceeded these frequencies were not considered as potential mutations, even if they were recorded as such in the Human Gene Mutation Database (HGMD). Finally, variants were searched in the HGMD by internet search engine [19,20]. Variants identified as possibly disease causing were confirmed by Sanger sequencing using a Big-Dye Terminator v3.1 Kit on an ABI3130xl automatic DNA sequencer system (Applied Biosystems, Carlsbad, CA). We analyzed the non-synonymous single nucleotide substitutions with PolyPhen 2 (Polymorphism Phenotyping) to predict the possible impact of amino acid substitutions on the structure and function of a protein [21].

In each patient’s report, parental testing was recommended and offered at no additional charge to further elucidate the significance of all confirmed, possibly disease causing variants. This parental testing was performed by Sanger sequencing as described above, and results were used to further interpret variants found in the proband.

### Quality control metrics

A variety of quality control metrics were used to ensure that patient runs were of the highest quality. These included an examination of the percentage of reads mapping to the human genome, the percentage of reads mapping to the targeted regions, the average read depth of targeted regions, and the percentage of targets with greater than an average of 20× coverage per base with a quality score of Q ≥ 30. An external control was included in the eighth lane of every sequencing run and consisted of a previously characterized HapMap sample (Coriell Institute, Camden, NJ).

## Results

### Sequencing and quality control

On average, 67.4% of reads aligned to the human genome, and 61.2% aligned to the targeted regions. Average coverage of the targeted regions was 147× per base, and on average, 95.6% of targets had greater than 20× coverage per base with a score of Q ≥ 30. Internal and external controls were used to verify the quality of each sequencing run. In the external HapMap control, we identified on average 164.7 variants per run (95.7%) of the 172 previously characterized SNPs in this HapMap sample within the targeted regions. We also used a panel of 200 SNPs with high minor allele frequencies in the first 20 patients as an internal sensitivity control for all samples, and we identified an average of 194.3 (94.7%) of the SNPs in each patient.

### Variants in targeted genes

On average, we found 553 variants per patient in the targeted regions. Synonymous single nucleotide substitutions were the most common, followed closely by intronic/UTR (limited to 20 nucleotides from the exon boundary), and missense single nucleotide substitutions (Additional file 2: Figure S1). Nonsense and canonical splice-site single nucleotide substitutions and insertion/deletion variants were far less common among patients. After all variants were compared to public SNP databases, and an internal SNP database, most were eliminated from consideration because they were highly prevalent in these cohorts. Variants were also searched for in HGMD, and several observed variants were considered to be misannotated as pathogenic, given their high MAF value. Following this comparison, on average 6.5 variants of interest were identified per patient. Most of these were single heterozygous variants of unknown significance in genes with AR disease inheritance.

### Suspected disease-causing alterations and variants of unknown significance in patients

We identified variants considered to be possibly disease-causing based in twelve genes in thirteen patients (Table 2). These variants were suspected to be disease-causing based on their low MAF, and their appropriate segregation within parental samples; eleven of the 21 variants had also been published as disease-causing mutations (Table 2). While we suspect that these variants are disease-causing, the true significance of each variant cannot be determined without functional studies. Even when variants have been published as disease-causing mutations, it is known that these variants can later be classified as benign. In all clinical reports, we indicate that clinical correlation is required to confirm the molecular diagnosis and that the results of this test are not intended to be used as the sole means for patient diagnosis or patient management decisions and must be used in conjunction with the patient’s clinical history and any previous analysis of appropriate family members.

**Table 2:** List of disease with possible disease-causing alterations detected in patients

**Case**	**Gene**	**Disease (OMIM)**	**Mode**	**Nucleotide change **^***a***^	**Protein change**	**dbSNP rs ID**	**MAF (%)**	**HGMD ID**	**Parental result/patient’s sex**	**Polyphen2 prediction**^**b**^
***RCC subunit and assembly factor***										
1	*NDUFS2*	Mitochondrial complex 1 deficiency (252010)	AR	NM_004550.4 455_457delCTC	Ser152del	---	---	---	Paternal	---
				875 T > C	Met292Thr	150667550	<0.01	CM094573	Maternal	Possibly damaging
2	*NDUFAF5*	Mitochondrial complex 1 deficiency (252010)	AR	NM_024120.4 164A > G	Gln55Arg	---	---	---	Maternal	Probably damaging
				327 + 3A > G		---	---	---	Paternal	
***mtDNA synthesis, transcription, translation, mitochondrial biogenesis and dynamic***										
3*	*MTFMT*	Leigh syndrome (256000)	AR	NM_139242.3 626G > A	Ser209Leu	201431517	0.1	CS117162	Paternal	Possibly damaging-
				998G > C	Ser333Ter	---	---		Maternal	---
4	*TYMP*	Mitochondrial DNA depletion syndrome 1 (MNGIE type) (603041)	AR	NM_001953.3 1160G > A homozygote	Gly387Asp	---	---	CM055161	---	Probably damaging
***Mitochondrial enzymes***										
5*	*ETFB*	Glutaric acidemia IIB (231680)	AR	NM_001014763.1 235G > A	Val79Ile	140608276	0.4	---	Maternal	Benign
				565C > T	Arg189Cys	147353781	0.3	---	Paternal	Possibly damaging
6	*PANK2*	HARP syndrome (607236) / Neurodegeneration with brain iron accumulation 1 (234200)	AR	NM_153638.2 137A > T	Asp46Val	148036492	0.2	---	Maternal	Benign
				1561G > A	Gly521Arg	137852959	<0.02	CM014248	Paternal	Probably damaging
7*	*PCK2*	PEPCK deficiency, mitochondrial (261650)	AR	NM_004563.2 731G > A	Arg244Gln	---	<0.02	---	Not in mother; father not sequenced	Possibly damaging
				1756G > A	Gly586Ser	61737098	0.4	---	Maternal	Possibly damaging
8*	*OTC*	Ornithine transcarbamylase deficiency (311250)	X-linked	NM_000531.5 298 + 5G > C hemizygote	---	72554348	0.2	CS063357	Male	---
***Other genes that affect mitochondrial function or that cause similar clinical phenotypes***										
9*/13	*SPAST*	Spastic paraplegia 4 (182601)	AD	NM_014946.3 1625A > G	Asp542Gly	142053576	<0.05	CM054864	---	Benign
10	*CLN6*	Ceroid lipofuscinosis (CLN) 6 (601780)/ CLN Kufs type (204300)	AR	NM_017882.2 278 T > C	Thr93Met	150001589	<0.04	CM120905	Maternal	Probably damaging
				775G > A	Gly259Ser	150363441	<0.02		Paternal	Probably damaging
11	*SLC12A3*	Gitelman syndrome (263800)	AR	NM_000339.2 322C > T	Arg108Trp	---	---	CM117057	---	Possibly damaging
				965C > T	Ala322Val	142679083	0.5	CM117024	---	Benign
12*	*SLC3A1*	Cystinuria (220100)	AR	NM_000341.3 241C > T	Arg81Cys	149813423	<0.02	CM090053	Maternal	Benign
				1400 T > C	Met467Thr	121912691	0.4	CM941280	Paternal	Benign

^a^ All variants listed were heterozygous, except where notated otherwise.

^b^ Prediction by Polyphen2 HumVar model.

* cases for which abnormal RCC activity and/or muscle pathology were reported.

The variants identified as possibly disease-causing were located in the genes *MTFMT* (Leigh syndrome) [22,23], *NDUFS2* (Mitochondrial complex I deficiency) [24], *NDUFAF5* (Mitochondrial complex I deficiency) [25], *ETFB* (Glutaric acidemia IIB) [26], *PANK2* (Neurodegeneration with brain iron accumulation) [27], *PCK2* (Phosphoenolpyruvate carboxykinase deficiency) [28], *SLC3A1* (Cystinuria) [29], *TYMP* (Mitochondrial neurogastrointestinal encephalopathy) [30], *OTC* (Ornithine transcarbamylase deficiency) [31], *SPAST* (Spastic paraplegia 4) [32], *SLC12A3* (Gitelman syndrome) [33] and *CLN6* (Ceroid lipofuscinosis 6) [34]. *CLN6* and other non mitochondrial nuclear genes were included in our panel as the associated clinical presentation can be easily confused with mitochondrial disease. PolyPhen-2 predictions for these variants are listed in Table 2. These predictions are provided for reference only, and were not used in the classification of variants, as *in silico* prediction models are known to not always be accurate [35-37].

Variants of unknown significance were identified in genes previously implicated in mitochondrial disorders or in conditions that present with a similar phenotype in 67 patients (Additional file 3: Table S2 variants without family segregation data, Additional file 4: Table S3 and Additional file 5: Table S4 variants with decreased suspicion after parental testing). To elucidate the significance of these novel or rare variants, parental testing was recommended. However, we received parental samples for only 30 of the 83 cases for which testing was suggested. Of the 15 potentially compound heterozygous variants, we have been able to confirm that eight of them were in *trans*, therefore consistent with AR disease inheritance (Table 2).

With regard to the genes with AD disease inheritance, 19 of 20 variants tested in parental samples were identified in one of the patient’s parents (Additional file 5: Table S4) and a *KIF1B* variant was tested only in the patient’s father as a maternal sample was unavailable. If a variant was found in both the patient and the patient’s presumably healthy parents, the suspicion of its pathogenicity was greatly reduced; however, it is still possible that incomplete penetrance or differences in expression could explain finding a pathogenic mutation in a healthy parent.

One known mutation in the *OTC* gene (Table 2) and seven variants of unknown significance in six genes were detected with XL disease inheritance including *DCX* (lissencephaly), *PDHA1* (PDH deficiency), *ABCD1* (X-linked adrenoleukodystrophy), *MECP2*, *CDKL5* (Rett syndrome), and *SLC6A8* (creatine transport defect) (Additional file 1: Tables S2 and Additional file 4: Table S3). We received parental samples to test only two of these alterations (*CDKL5*, *DCX*) (Additional file 4: Table S3). Both variants were found to be in the patient’s mothers, thereby reducing the likelihood of these variants being pathogenic. Nevertheless, the *CDKL5* variant was still suspicious due to the clinical phenotype of the patient, since the referring physician considered his disease to be consistent with Angelman syndrome, and previously ruled out *UBE3A* methylation and sequence defects. Skewed X-inactivation in the patient’s mother could explain the presence of the variant in the healthy mother, if it is indeed pathogenic. It is also possible that this particular variant only mildly affects protein function which can be overcome in a female, but may be detrimental in a male.

## Discussion

Diagnosis of mitochondrial disease by NGS of targeted gene panels has been explored on a research basis over the past years [2,10,11,15]. This work has proven to be highly effective in diagnosing patients, and identifying new disease genes [2,11,15,22,38-41]. This technology is providing insights into the genetic basis of diseases that often remain undiagnosed even after extensive clinical workup [42]. The reliability of our approach has been previously demonstrated from our earlier research studies [10,15], and is similar to the approaches used for the diagnosis of mitochondrial disorders and other inborn errors of metabolism in other laboratories [2,40,43,44]. This approach has very high analytical sensitivity and specificity as demonstrated by our quality control metrics and use of internal and external controls. The clinical sensitivity and specificity of the approach are less easily defined. Our classification approach utilizes conservative minor allele frequency cut-offs to insure that disease causing variants are not removed from consideration. While this does allow for many rare, benign polymorphisms to be reported as variants of unknown significance, we must rely on the patient’s physician to determine if these variants fit the patient’s clinical presentation.

In this clinical study, patient samples were submitted by physicians, most of whom were experts in the field of mitochondrial medicine and ordered this test because other diagnostic avenues had been unsuccessful and mitochondrial disease was still suspected. As a result, this patient cohort is more representative of the wide variety of patients that are considered to have suspected mitochondrial disease in comparison to the research studies of the past.

Our data highlights the great underlying genetic heterogeneity of suspected mitochondrial disease, but also the difficulty that even experienced physicians endure when attempting to define mitochondrial disorders. Of note, possible disease-causing variants, confirmed by parental samples analysis, were found in only one gene that encodes an oxidative phosphorylation subunit of complex I (*NDUFS2*) and one gene for the assembly of the complex I (*NDUFAF5*). In a total of 148 patients we analyzed, there was only one additional gene *SCO2* in the category of primary mitochondrial disease causative genes, with variants that we classified as of unknown significance as we did not receive parental samples, but most likely associated with disease.

Our results indicate that the majority of patients that cannot obtain a molecular diagnosis by traditional methods (RCC enzyme assay), have disease that is probably not the result of direct impairment of energy production as we observed in our previous study [15]. Instead, their disease may be caused by secondary inhibition of proper mitochondrial function [30,45,46]. This observation is consistent with other studies that utilized targeted sequencing on patients with biochemically confirmed defects [2,15,23]. Other NGS studies have also indicated that suspected “mitochondrial patients” were actually affected by conditions caused by mutations in genes that don’t impair mitochondria function [47,48]. While we can’t entirely exclude that some patients may have mutations in mtDNA, nuclear genes are primarily causative for pediatric cases, which are the majority of the patients studied here [1]. The requisition forms for the patients with variants listed in the Tables indicated that 35 of them had mtDNA sequenced with either normal or inconclusive results.

From our observation, it appears that the clinical differential diagnosis for mitochondrial disease could be even more challenging, as the diagnostic spectrum is much wider than previously thought. Some of our patients could have been diagnosed by standard biochemical or genetic studies, without NGS sequencing. Unfortunately, the lack of clinical phenotypes that are specific to these diseases causes physicians to have a difficult time identifying which biochemical studies should be ordered for each case. A good example is represented by the patient affected by CLN6, a lysosomal storage disorder in which the clinical presentations can easily mislead to focus on mitochondria, leading to muscle biopsy and RCC assay.

While it is undoubtedly important to perform family segregation analysis for the accurate and appropriate interpretation of novel variants, we could not obtain more parental samples in this study as some of parents were not involved in patient’s care. We can nevertheless consider that even when definitive diagnoses cannot be made in the absence of family segregation supporting data, the results can still guide physical examinations or additional laboratory tests to look for signs specific to the diseases for which possibly pathogenic variants were found. Some examples include *GAA* (Pompe disease), *HLCS* (Holocarboxylase synthetase deficiency), *ABCD1* (X-linked adrenoleukodystrophy), *PC* (Pyruvate carboxylase deficiency), *UBE3A* (Angelman syndrome), *ASL* (Argininosuccinic aciduria), or *ZFYVE26* (Spastic paraplegia 15) (Additional file 3: Table S2). Presumptive diagnosis can prompt recommended interventions, including diet modification for certain patients such as for ornithine transcarbamylase deficiency, one of the urea cycle disorders. It is unfortunate that we cannot provide further details on any follow-up biochemical tests that were recommended to the referring physicians. The nature of clinical testing does not allow for us to regularly follow-up on patients tested and how our results affected their care.

Our results highlight the need to have better means to define the significance of variants found. For instance, the variants of unknown significance in genes with AD inheritance were also found in majority of one of presumably healthy parents. While this cannot entirely rule out the possibility of pathogenic involvement, only functional studies can truly assess the significance of these variants. This problem is an even larger issue when whole exome or whole genome sequencing data are interpreted.

Of interest, we have observed that many patients had possibly disease-causing variants in multiple genes. This suggests that some patients with suspected mitochondrial disease may be caused by oligogenic factors, where many mildly dysfunctional proteins may be insufficient to cause disease alone, but when in combination can cause disease in a patient. If this is possible, it even further complicates the molecular diagnosis of these patients and extended studies will be necessary to explore this possibility. We also observed a substantial number of VUS for Charcot-Marie-Tooth (CMT) disease, spinocerebellar ataxia or spastic paraplegia related genes. While these variants still require parental samples analysis to determine the clinical significance, these conditions may be an emerging group of mitochondrial disorders as supported by recent publications with evidence of mitochondrial dysfunction [49,50].

## Conclusions

Our study revealed that a broader diagnostic spectrum including neurodegenerative disorders or myopathy need to be considered during the clinical work-up of suspected mitochondrial patients and in developing future diagnostic tests. It appears that the current standard diagnostic algorithm along with the definition of mitochondrial disorders may require substantial revision to ultimately improve patient quality care. The clinical sensitivity of NGS assays for mitochondrial disorders will be further improved by targeting more genes and ultimately by whole exome sequencing with adequate coverage in the near future.

## Competing interests

J. DaRe is an employee of, and holds stock options for Transgenomic, Inc. Seattle Children’s Research Institute receives a licensing fee for all NuclearMitome tests run through Transgenomic, Inc. Transgenomic, Inc is financing the article-processing charge for this manuscript.

## Authors’ contributions

VV and JTD equally contributed to this manuscript. JTD drafted the manuscript and analyzed and interpreted the data. VV selected and designed the targets capture, performed experiments, drafted the manuscript, analyzed and interpreted the data. JP analyzed the data. TT performed experiments. SHH conceived of the study concept, drafted the manuscript, and analyzed and interpreted the data. All authors read and approved the final manuscript.

## Pre-publication history

The pre-publication history for this paper can be accessed here:

http://www.biomedcentral.com/1471-2350/14/118/prepub

## Supplementary Material

Additional file 1: Table S1 List of Genes for Nuclear Mitome Test (447 genes). Click here for file

Additional file 2: Figure S1 Average number of variants per patients in targeted regions. Click here for file

Additional file 3: Table S2 List of disease with variants of unknown significance (VUS) detected in patients for which parental testing results are not available. Click here for file

Additional file 4: Table S3 Variants of unknown significance identified in autosomal dominant and X-linked genes with decreased suspicion after parental testing. Click here for file

Additional file 5: Table S4 Variants of unknown significance identified in autosomal recessive genes with decreased suspicion after parental testing. Click here for file
