# Supplementary material for: Targeted exome sequencing for mitochondrial disorders reveals high genetic heterogeneity
Source: BMC Med Genet. 2013 Nov 11;14:118. doi: 10.1186/1471-2350-14-118 (PMC3827825; doi:10.1186/1471-2350-14-118)
Supplement: Additional file 1: Table S1 — List of Genes for Nuclear Mitome Test (447 genes). [file 1471-2350-14-118-S1.pdf]

**Table S1. List of Genes for Nuclear Mitome Test (447 genes)**

**Respiratory chain complexes subunits (89):**

**Complex 1 -** *NDUFA1, NDUFA10, NDUFA11, NDUFA12, NDUFA13*, NDUFA2, NDUFA3*, NDUFA4*, NDUFA4L2, NDUFA5*, NDUFA6*, NDUFA7*, NDUFA8*, NDUFA9, NDUFAB1*, NDUFB1*, NDUFB10*, NDUFB11*, NDUFB2*, NDUFB3, NDUFB4*, NDUFB5*, NDUFB6*, NDUFB7*, NDUFB8*, NDUFB9, NDUFC1*, NDUFC2*, NDUFS1, NDUFS2, NDUFS3, NDUFS4, NDUFS5*, NDUFS6, NDUFS7, NDUFS8, NDUFV1, NDUFV2, NDUFV3**

**Complex 2 -** *SDHA, SDHB, SDHC, SDHD*

**Complex 3**-*CYC1*, CYCS, UQCR10*, UQCR11*, UQCRB, UQCRC1*, UQCRC2, UQCRFS1*, UQCRH*, UQCRQ*

**Complex 4 -** *COX4I1*, COX4I2, COX5A*, COX5B*, COX6A1*, COX6A2*, COX6B1, COX6B2*, COX6C*, COX7A1*, COX7A2*, COX7A2L*, COX7B, COX7B2*, COX7C*, COX8A*, COX8C**

**Complex 5 -** *ATP5A1*, ATP5B*, ATP5C1*, ATP5D*, ATP5E, ATP5F1*, ATP5G1*, ATP5G2*, ATP5G3*, ATP5H*, ATP5I*, ATP5J*, ATP5J2*, ATP5L*, ATP5O*, ATP5S*, ATPIF1*, C14orf2*, USMG**

**Respiratory chain complexes assembly factors (29):**

**Complex 1-** *AIFM1, AIFM2*, ECSIT*, FOXRED1, NDUFAF1, NDUFAF2, NDUFAF3, NDUFAF4, NDUFAF5, NDUFAF6, NUBPL*

**Complex 2 -** *SDHAF1, SDHAF2*

**Complex 3 -** *BCS1L*

**Complex 4 -** *CMC1*, CMC2*, COX10, COX11*, COX15, COX17*, COX18*, COX19*, OXA1L*, SCO1, SCO2, SURF1*

**Complex 5 -** *ATPAF1, ATPAF2, TMEM70*

**Mitochondrial DNA synthesis, transcription, translation, mitochondrial biogenesis and dynamic (71):**

*AARS2, AFG3L2, APEX2, C10orf2, C12orf65, CARS2*, DARS2, DCX, DGUOK, DMPK, DNM1L, DNM2, EARS2, EIF2AK3, FARS2, FASTKD2, FOXG1, GARS, GFM1, HARS2, HSPD1, IARS2*, KARS, KIF1B, KIF5A, LARS2, LETM1, LRPPRC, MAPT, MARS2, MFN2, MPV17, MRPS16, MRPS22, MTFMT, MTO1, MTPAP, MUTYH, NARS2*, NEFL, NKX2-1, OPA1, OPA3, PARS2*, PDX1, POLG, POLG2, PUS1, RARS2, REEP1, RNASEH2A, RNASEH2B, RNASEH2C , RRM2B, SARS2, SPG20, SPG7, SUCLA2, SUCLG1, TACO1, TARS2*, TK2, TOP1MT, TRMU, TSFM, TUFM,TYMP, VARS2*, WARS2*, WFS1, YARS2*

**Mitochondrial carriers (17):**

*ABCB7, DNAJC19, SLC25A12, SLC25A13, SLC25A15, SLC25A16, SLC25A19, SLC25A20, SLC25A22, SLC25A3, SLC25A38, SLC25A4, STAR, TIMM8A, UCP1, UCP , UCP3*

**Mitochondrial enzymes (114):**

*AASS, ABAT, ACAD9, ACADL, ACADM, ACADSB, ACADVL, ACAT1, ACSF3, ADCK3, AK2, ALAS2, ALDH18A1, ALDH4A1, ALDH5A1, ALDH6A1, AMACR, AMT, ARG1, ASL, ASS1, AUH, BCKDHA, BCKDHB, C7orf10, COQ2, COQ3*, COQ4, COQ5*, COQ6, COQ7*, COQ9, CPOX, CPS1, CPT1A, CPT2, CRLS1, CYB5R3, CYP11A1, CYP11B1, CYP11B2, CYP27A1, CYP27B1, D2HGDH, DBT, DECR1, DLAT, DLD, DLST, DMGDH, ETFA, ETFB, ETFDH, ETHE1, FECH, FH, FXN, GATM, GCDH, GCK, GCSH, GFER, GK, GLDC, GLRX5, GLUD1, GPD2, HADH, HADHA, HADHB, HCCS, HK1, HMGCL, HMGCS2, HSD17B10, HSD3B2, IDH2, IVD, MAOA, MCCC1, MCCC2, ME2, MLYCD, MMAA, MMAB, MMACHC, MMADHC, MTRR, MUT, NAGS, OAT, OGDH, OXCT1, OTC, PANK2, PARL, PC, PCCA, PCCB, PCK2, PDHA1, PDHB, PDHX, PDP1, PDSS1, PDSS2, PHYH , PNKD, PPOX, PRODH, SUOX, TAZ, UNG, XPNPEP3*

**Other genes that affect mitochondrial function or that cause similar clinical phenotypes (127):**

*ABCD1, ABHD5, ACSL4, AGXT, AKR1D1, ALG1, ALG12, ALG2, ALG3, ALG6, ALG8, ALG9, APTX, ARMS2, ATL1, ATM, ATP7B, ATXN10, ATXN7, B4GALT1, BRAF, BTD, CAPN3, CAV3, CDKL5, CHKB, CISD2, CLN3, CLN5, CLN6, CLN8, COG1, COG7, COG8, COQ10A*, COQ10B*, CRYAB, CTNS, CTSD, CYB5A, CYBA, CYBB, CYP7B1, DOLK, DPAGT1, DPM1, DPM3, ELOVL4, FA2H, FGF14, GAA, GAD1, GAMT, GDAP1, GLA, GNPAT, GPHN, HAX1, HFE, HLCS, ISCU, ITPR1, KCNC3, KCNJ11, KIAA0196, KIAA0226, LMBRD1, MECP2, MFSD8, MGAT2, MOCS1, MOCS2, MOGS, MPDU1, MPI, MTHFD1, MYH7, NIPA1, NPC1, NPC2, PAFAH1B1, PAR1, PEX13, PHB, PLP1, PMM2, PNPLA2, PNPLA3, PPM1B, PPT1, PREPL, PRKCG, RFT1, RMRP, RYR1, SAMHD1, SCN1A, SGCD, SLC12A3, SLC16A2, SLC19A2, SLC22A5, SLC29A3, SLC2A1, SLC2A10, SLC33A1, SLC35A1, SLC35C1, SLC3A1, SLC52A1, SLC6A8, SLC7A13*, SLC7A9, SOD1, SPAST, SPG11, SPTBN2, TAT, TMEM126A, TMEM126B, TPM2, TPP1, TTBK2, UBE3A, UROS, XDH, ZFYVE26*

*candidate genes
