# Supplementary figures and images for: Targeted exome sequencing for mitochondrial disorders reveals high genetic heterogeneity
Source: BMC Med Genet. 2013 Nov 11;14:118. doi: 10.1186/1471-2350-14-118 (PMC3827825; doi:10.1186/1471-2350-14-118)

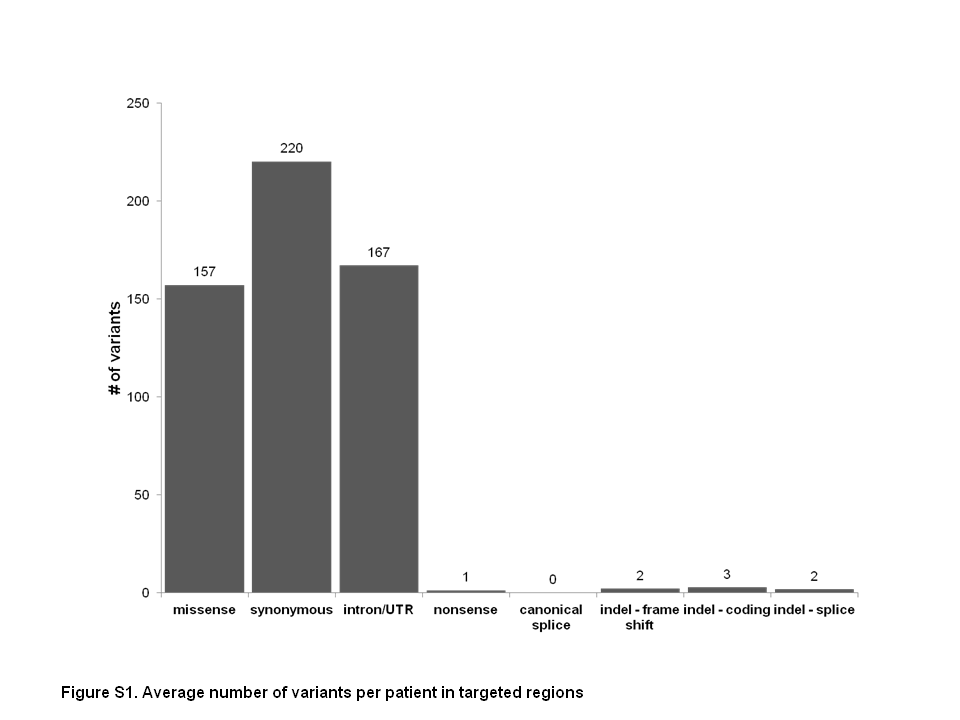

Supplement: Additional file 2: Figure S1 — Average number of variants per patients in targeted regions. [file 1471-2350-14-118-S2.tiff]
