# Supplementary material for: Targeted exome sequencing for mitochondrial disorders reveals high genetic heterogeneity
Source: BMC Med Genet. 2013 Nov 11;14:118. doi: 10.1186/1471-2350-14-118 (PMC3827825; doi:10.1186/1471-2350-14-118)
Supplement: Additional file 3: Table S2 — List of disease with variants of unknown significance (VUS) detected in patients for which parental testing results are not available. [file 1471-2350-14-118-S3.docx]

**Table S2. List of disease with variants of unknown significance (VUS) detected in patients for which parental testing results are not available**

| **Case** | **Gene** | **Disease (OMIM)** | **Inheritance mode** | **Nucleotide Change** *^a^* | **Protein Change** | **dbSNP rs ID** | **MAF (%)** | **HGMD ID** | **Patient’s sex** | **Polyphen2 Prediction^b^** |
| --- | --- | --- | --- | --- | --- | --- | --- | --- | --- | --- |
|  | ***RCC Assembly Factors*** | | | | | | | | | |
| *14* | *SCO2* | Cardioencephalomyopathy (604377)/ Mitochondrial complex IV deficiency (220110) | AR | NM_005138.2 401_402delCT | Pro134fs | --- | --- | --- |  | --- |
|  |  |  |  | 703G>A | Gly235Ser | --- | --- | --- |  | Probably damaging |
|  | ***mtDNA synthesis, transcription, translation, mitochondrial biogenesis and dynamic*** | | | | | | | | | |
| *15** | *DNM1L* | Encephalopathy due to defective mitochondrial peroxisomal fission (614388) | AD | NM_012062.3 297+14C>G | --- | --- | --- | --- |  |  |
| *16* |  |  |  | 1648G>A | Ala550Thr | --- | --- | --- |  | Benign |
| *17** |  |  |  | 1834A>T | Ile612Phe | 138133550 | 0.1 | --- |  | Benign |
| *18* | *DNM2* | Charcot-Marie-Tooth disease (CMT)DIB/CMT2M (606482) | AD | NM_001005360.2 778C>G | Leu260Val | 145607989 | <0.01 | --- |  | Benign |
| *19** | *DMPK* | Myotonic dystrophy 1 (160900) | AD | NM_001081563.1 37G>A | Val13Met | --- | --- | --- |  | Benign |
| *20* |  |  |  | 185C>G 1866C>A both seen in one patient | Pro62Arg His622Gln | --- --- | --- --- | --- --- |  | Benign  Benign |
| *21* |  |  |  | NM_004409.3 1477C>T | Arg493Cys | 78771765 | 0.1 | --- |  | Benign |
| *22* |  |  |  | 1840G>A | Gly614Ser | --- | --- | --- |  | Benign |
| *23* | *GARS* | CMT2D (601472)/Distal motor neuropathy V (600794) | AD | [NM_002047.2](http://www.ncbi.nlm.nih.gov/entrez/viewer.fcgi?val=NM_002047.2) 401A>G | Tyr134Cys | --- | --- | --- |  | Probably damaging |
| *24** |  |  |  | 843G>A | Met281Ile | --- | --- | --- |  | Probably damaging |
| *25* | *KIF1B* | CMT2A1 (118210) | AD | [NM_015074.3](http://www.ncbi.nlm.nih.gov/entrez/viewer.fcgi?val=NM_015074.3) 985G>C | Asp329His | --- | --- | --- |  | Probably damaging |
| *26* |  |  |  | 2321T>G | Phe774Cys | --- | --- | --- | Not Paternal**^b^** | Probably damaging |
| *27* |  |  |  | 2427G>A | Met809Ile | --- | --- | --- |  | Benign |
| *28** |  |  |  | 2537+3A>G | --- | 200684032 | 0.1 | --- |  |  |
| *29** |  |  |  | 2902G>T | Ala968Ser | --- | --- | --- |  | Benign |
| *30* |  |  |  | 3121+9A>G | --- | 149566646 | 0.1 | --- |  |  |
| *31* |  |  |  | NM_183416.3 2488T>G | Leu830Val | --- | --- | --- |  | Possibly damaging |
| *11* |  |  |  | NM_183416.3 3322C>T | Gln1108Ter | --- | --- | --- |  |  |
| *32* | *KIF5A* | SPG10 (604187) | AD | [NM_004984.2](http://www.ncbi.nlm.nih.gov/entrez/viewer.fcgi?val=NM_004984.2) 2927C>T | Thr976Ile | 139801016 | 0.2 | --- |  | Benign |
| *9** |  |  |  | 2943delG | Ser978fs | --- | --- | --- |  |  |
| *33* | *MAPT* | Dementia, frontotemporal, with or without parkinsonism (600274)/ Pick disease (172700)/ Supranuclear palsy, progressive (601104)/ Supranuclear palsy, progressive atypical (260540)/ Tauopathy and respiratory failure | AD | [NM_005910.5](http://www.ncbi.nlm.nih.gov/entrez/viewer.fcgi?val=NM_005910.5) 715G>A | Ala239Thr | 63750096 | 0.1 | --- |  | Benign |
| *31* | *NEFL* | CMT1F (607734)/CMT2E (607684) | AD | NM_006158.3 1610A>G | Gln537Arg | --- | --- | --- |  | Benign |
| *32* | *OPA3* | Costeff optic atrophy (2585010/Optical atrophy and cataract (165300) | AR/AD | [NM_001017989.2](http://www.ncbi.nlm.nih.gov/nuccore/NM_001017989.2)  256G>C | Glu86Gln |  |  |  |  |  |
| *33* | *C10orf2* | Mitochondrial DNA depletion syndrome 7 (hepatocerebral type) (271245) /Progressive external ophthalmoplegia, autosomal dominant, 3 (609286)/Infantile-onset spinocerebellar ataxia (IOSCA) | AR/AD | NM_021830.4 938G>A | Arg313Gln | --- | --- | --- |  | Probably damaging |
| *34** | *DCX* | Lissencephaly, X-linked /Subcortical laminal heteropia, X-linked (300067) | X-linked dominant | NM_000555.3 151A>G | Met51Val | --- | --- | --- | Female | Benign |
| *35* |  |  |  | NM_178153.2 809-3C>T | --- | --- | --- | --- | Female | --- |
| *36** |  |  |  | 856G>T | Ala286Ser | 149495971 | <0.01 | --- | Male | Benign |
| *37* | *RRM2B* | Mitochondrial DNA depletion syndrome 8B (MNGIE type) (612075)/Progressive external ophthalmoplegia with mitochondrial DNA deletions, autosomal dominant, 5 (613077) | AR/AD | NM_015713.4 544A>G | Thr182Ala | 147315735 | <0.01 | -- |  | Benign |
|  | ***Mitochondrial enzymes*** | | | | | | | | | |
| *38* | *IDH2* | D-2-hydroxyglutaric aciduria 2 (613657) | AD | NM_002168.2 844A>G | Lys282Glu | --- | --- | --- |  | Benign |
| *28* | *PNKD* | Paroxysmal nonkinesigenic dyskinesia (118800) | AD | NM_015488.4 265G>A | Gly89Arg | 147259983 | 0.2 | --- |  | Probably damaging |
| *39* | *PPOX* | Porphyria variegata (176200) | AD | NM_000309.3 1361T>C | Val454Ala | --- | --- | --- |  | Possibly damaging |
| *40* | *PC* | Pyruvate carboxylase deficiency (266150) | AR | NM_000920.3 616G>T | Val206Leu | 147945506 | 0.3 | --- |  | Benign |
|  |  |  |  | 2223+20G>A | --- | --- | --- | --- |  | ---- |
| *33*/41* | *PDHA1* | Leigh syndrome, X-linked (308930)/Pyruvate dehydrogenase E1-alpha deficiency (312170) | X-linked dominant | NM_000284.3 899+12G>A^c^ | --- | --- | --- | --- | Female | --- |
|  | ***Other genes that affect mitochondrial function or that cause similar clinical phenotypes*** | | | | | | | | | |
| *42* | *ATL1* | Sensory neuropathy type ID (613708)/ SGP3A (182600) | AD | NM_015915.4 1551+11C>G | --- | --- | --- | --- |  |  |
| *43** | *BRAF* | Noonan syndrome 6 (613706) | AD | NM_004333.4 466A>G | Ile156Val | --- | --- | --- |  | Benign |
| *44** | *SPTBN2* | Spinocerebellar ataxia (SCA) 5 (600224) | AD | NM_006946.2 1654-13G>A | --- | 200980512 | 0.2 | --- |  |  |
| *18* |  |  |  | 3116G>A | Arg1039Gln | 148826890 | 0.2 | --- |  | Benign |
| *29** |  |  |  | 5894_5896delCCT | Ser65del | --- | --- | --- |  |  |
| *37* | *UBE3A* | Angelman syndrome (105830) | AD | NM_130838.1 926A>G | Asn309Ser | --- | --- | --- |  | Benign |
| *45* | *ATXN7* | SCA7 (164500) | AD | NM_000333.3 187G>T | Gly63Cys | --- | --- | --- |  | Benign |
| *46* | *TTBK2* | SCA11 (604432) | AD | NM_173500.3 3172T>C | Ser1058Pro | --- | --- | --- |  | Benign |
| *35* |  |  |  | NM_173500.3 3539G>A | Ser1180Asn | 200469913 | 0.1 | --- |  | Probably damaging |
| *47* | *ITPR1* | SCA15 (606658) | AD | NM_002222.5 4349C>T | Thr1450Ile | 188558398 | 0.1 | --- |  | Probably damaging |
| *48** |  |  | AD | NM_001168272.1 5118C>A | Asn1706Lys | 201937660 | --- | --- |  | Benign |
| *22* | *KIAA0196* | Spastic paraplegia (SPG) 8 (603563) | AD | NM_014846.3 2273T>C | Ile758Thr | 184833599 | 0.1 | --- |  | Probably damaging |
| *49* |  |  |  | 3296T>C | Ile1099Thr | --- | --- | --- |  | Benign |
| *50* | *ZFYVE26* | SPG15 (270700) | AR | [NM_015346.3](http://www.ncbi.nlm.nih.gov/entrez/viewer.fcgi?val=NM_015346.3) 274-3T>C | --- | --- | --- | --- |  |  |
|  |  |  |  | 1597G>A | Ala533Thr | --- | --- | --- |  | Benign |
| *36** | *SLC33A1* | Congenital cataracts, hearing loss, and neurodegeneration (614482)/SPG42 (612539) | AR/AD | NM_004733.3 136G>A | Glu46Lys | 149571533 | 0.1 | --- |  | Benign |
| *51* | *SLC2A1* | GLUT1 deficiency syndrome 1 and 2 (606777) (612126) | AD | NM_006516.2 764A>C | Lys255Thr | 5811 | 0.2 | --- |  | Benign |
| *52* |  |  |  | NM_006516.2 1408G>C | Gly470Arg | --- | --- | --- |  | Benign |
| *53* | *GAA* | Glycogen storage disease II (232300) | AR | NM_000152.3 -32-13T>G | --- | 199951626 | 0.3 | CS941489 |  |  |
|  |  |  |  | 1552-13G>A | --- | 111261964 | 0.4 | --- |  |  |
| *16* | *HLCS* | Holocarboxylase synthetase deficiency (253270) | AR | NM_000411.6 1633dupT | Asn511fs | --- | --- | --- |  |  |
|  |  |  |  | 1921G>A | Val641Met | 150665851 | 0.3 | --- |  | Benign |
| *32* | *ASL* | Argininosuccinic aciduria (207900) | AR | NM_000048.3 451C>T | Arg151Cys | --- | --- | --- |  | Benign |
|  |  |  |  | 811G>A | Val271Met | --- | --- | --- |  | Probably damaging |
| *38* | *ABCD1* | Adrenoleukodystrophy/ Adrenomyeloneuropathy (300100) | X-linked | NM_000033.3 100C>T | Pro34Ser | --- | --- | --- | Male | Possibly damaging |
| *54** | *MECP2* | Rett syndrome (312750)/ Rett syndrome, preserved speech variant (312750) | X-linked | NM_004992.3 608C>T | Thr203Met | 61749720 | <0.01 | --- | Female | Benign |
| *36** | *SLC6A8* | Creatine deficiency syndrome (300352) | X-linked | NM_005629.3 1646T>G | Asn549Thr | --- | --- | --- | Male | Benign |
| *55* | *RYR1* | Central core disease (117000)/Minicore myopathy with external ophthalmoplegia (255320)/ Neuromuscular disease, congenital, with uniform type 1 fiber (117000) | AR/AD | [NM_000540.2](http://www.ncbi.nlm.nih.gov/entrez/viewer.fcgi?val=NM_000540.2) 2645C>T | Ala882Val | 143701391 | 0.1 | --- |  | Possibly damaging |
| *45* |  |  |  | 2659G>A | Glu887Lys | --- | --- | --- |  | Probably damaging |
| *34** |  |  |  | 14305-3C>A |  |  |  |  |  |  |
| *35* | *KCNC3* | Spinocerebellar ataxia 13 (605259) | AD | NM_004977.2 188A>G | Asp63Gly |  |  |  |  | Benign |
| *56* |  |  |  | 884C>T | Ala295Val |  |  |  |  | Benign |
| *57** |  |  |  | 1984C>T | Arg662Cys |  |  |  |  | Possibly damaging |

^a^ All variants listed were heterozygous, except where notated otherwise. X-linked variants in male patients were hemizygous as expected.

^b^Prediction by Polyphen2 HumVar model

^c^This variant was seen in 2 patients

*cases for which abnormal RCC activity and/or muscle pathology was reported

Frame shifts mutations are designated by "fs" after the amino acid affected by the change
