# Supplementary material for: Targeted exome sequencing for mitochondrial disorders reveals high genetic heterogeneity
Source: BMC Med Genet. 2013 Nov 11;14:118. doi: 10.1186/1471-2350-14-118 (PMC3827825; doi:10.1186/1471-2350-14-118)
Supplement: Additional file 4: Table S3 — Variants of unknown significance identified in autosomal dominant and X-linked genes with decreased suspicion after parental testing. [file 1471-2350-14-118-S4.docx]

**Table S3. Variants of unknown significance identified in autosomal dominant and X-linked genes with**

**decreased suspicion after parental testing**

| **Case #** | **Gene** | **Nucleotide Change *^a^*** | **Protein Change** | **dbSNP rs ID** | **MAF (%)** | **HGMD ID** | **Parental result** | **Polyphen2 Prediction (HumVar)** |
| --- | --- | --- | --- | --- | --- | --- | --- | --- |
| *58* | *FGF14* | NM_004115.3 124G>T | Gly42Cys | 141304687 | 0.1 | --- | Paternal | Possibly damaging |
| *59* | *IDH2* | NM_002168.2 23T>C | Val8Ala | --- | --- | --- | Maternal | Benign |
| *60* | *ITPR1* | NM_002222.5 989G>A | Arg330Gln | 201804963 | 0.2 | --- | Maternal | Benign |
| *61* |  | 2979G>C | Gln993His | --- | --- | --- | Paternal | Benign |
| *62* | *KCNC3* | NM_004977.2 1978+3G>A | --- | --- | --- | --- | Paternal |  |
| *62** | *KIAA0196* | NM_014846.3 647C>T | Pro216Leu | 72720524 | 0.2 | --- | Maternal | Probably damaging |
| *63** |  | 2258G>A | Arg753His | --- | --- | --- | Paternal | Probably damaging |
| *64* | *MYH7* | NM_000257.2 4210G>A | Val1404Met | --- | --- | --- | Paternal | Probably damaging |
| *65** | *SCN1A* | NM_001165963.1 5858T>C | Leu1953Pro | --- | --- | --- | Maternal | Benign |
| *66** | *SLC2A1* | NM_006516.2 18+12G>T | --- | --- | --- | --- | Paternal |  |
| *67** | *SLC33A1* | NM_004733.3 1057G>T | Val353Phe | --- | --- | --- | Maternal | Benign |
| *64* | *SPTBN2* | NM_006946.2 406T>C | Met136Val | 150610657 | <0.01 | --- | Maternal | Possibly damaging |
| *68** |  | 1925G>A | Arg642Gln | --- | --- | --- | Maternal | Possibly damaging |
| *69** |  | 3671A>G | Asn1224Ser | 139077453 | 0.1 | --- | Maternal | Benign |
| *70* |  | 6802G>A | Val2268Met | --- | --- | --- | Maternal | Benign |
| *71* |  | 6929C>T | Ala2310Val | --- | --- | --- | Maternal | Probably damaging |
| *72* | *TTBK2* | NM_173500.3 2060G>T | Gly687Val | --- | --- | --- | Maternal | Probably damaging |
| *73* | *CDKL5* | NM_003159.2 2593C>A hemizygote | Gln865Lys | --- | --- | --- | Maternal (Male) | Benign |
| *74** | *DCX* | [NM_178153.2](http://www.ncbi.nlm.nih.gov/entrez/viewer.fcgi?val=NM_178153.2) 705+48A>G |  | 201805884 | 0.2 | CS024006 | Maternal (female) |  |

*^a^* All variants listed were heterozygous, except where notated otherwise

*cases for which abnormal RCC activity and/or muscle pathology was reported
