# Supplementary material for: Targeted exome sequencing for mitochondrial disorders reveals high genetic heterogeneity
Source: BMC Med Genet. 2013 Nov 11;14:118. doi: 10.1186/1471-2350-14-118 (PMC3827825; doi:10.1186/1471-2350-14-118)
Supplement: Additional file 5: Table S4 — Variants of unknown significance identified in autosomal recessive genes with decreased suspicion after parental testing. [file 1471-2350-14-118-S5.docx]

**Table S4. Variants of unknown significance identified in autosomal recessive genes with decreased suspicion after parental testing**

| **Case #** | **Gene** | **Nucleotide Change *^a^*** | **Protein Change** | **dbSNP rsID** | **MAF (%)** | **HGMD ID** | **Parental result** | **Polyphen2 Prediction (HumVar)** |
| --- | --- | --- | --- | --- | --- | --- | --- | --- |
| *70* | *NDUFAF6* | NM_152416.3 838G>A homozygote | Val280Ile | 61743028 | 0.4 | --- | Maternal Homozygote, Paternal Heterozygote | Benign |
| *59* | *EARS2* | NM_001083614.1 670G>A | Gly224Ser | 141129877 | 0.2 | CM123414 | Maternal | Probably damaging |
|  |  | 1412A>C | Lys471Thr | 137973249 | -- | --- | Maternal | Benign |
| *60* | *SLC12A3* | [NM_000339.2](http://www.ncbi.nlm.nih.gov/entrez/viewer.fcgi?val=NM_000339.2) 1928C>T | Pro643Leu | 140012781 | 0.2 | CM014405 | Maternal | Probably damaging |
|  |  | 2891G>A | Arg964Gln | 202114767 | 0.2 | --- | Maternal | Possibly damaging |
| *75* | *SUCLG1* | [NM_003849.3](http://www.ncbi.nlm.nih.gov/entrez/viewer.fcgi?val=NM_003849.3) 341T>C | Thr114Met | 201224138 | 0.1 | --- | Paternal | Possibly damaging |
|  |  | 566G>A | Met186Val | --- | --- | --- | Paternal | Benign |
| *58* | *SUOX* | [NM_000456.2](http://www.ncbi.nlm.nih.gov/entrez/viewer.fcgi?val=NM_000456.2) 1358G>A homozygote | Gly453Asp | 76537761 | 0.4 | --- | ---^b^ | Probably damaging |

*^a^* All variants listed were heterozygous, except where notated otherwise

*^b^* Parental testing was not performed, but symptomatic siblings were not homozygous and therefore this variant is not suspected to be the disease causing variant in the family.

*cases for which abnormal RCC activity and/or muscle pathology was reported
